# Lenalidomide treatment and prognostic markers in relapsed or refractory chronic lymphocytic leukemia: data from the prospective, multicenter phase-II CLL-009 trial

**DOI:** 10.1038/bcj.2016.9

**Published:** 2016-03-11

**Authors:** A Bühler, C-M Wendtner, T J Kipps, L Rassenti, G A M Fraser, A-S Michallet, P Hillmen, J Dürig, S A Gregory, M Kalaycio, T Aurran-Schleinitz, L Trentin, J G Gribben, A Chanan-Khan, B Purse, J Zhang, S De Bedout, J Mei, M Hallek, S Stilgenbauer

**Affiliations:** 1 Department of Internal Medicine III, Ulm University, Ulm, Germany; 2 Klinikum Schwabing, Academic Teaching Hospital of University of Munich, Munich, Germany; 3 Department I of Internal Medicine, University of Cologne, Cologne, Germany; 4 University of California San Diego Moores Cancer Center, La Jolla, CA, USA; 5 McMaster University, Juravinski Cancer Centre, Hamilton, ON, Canada; 6 Hospices civils de Lyon, Centre Hospitalier Lyon Sud, Lyon, France; 7 St James's Institute of Oncology, Leeds, UK; 8 University Hospital Essen, Essen, Germany; 9 Rush University Medical Center, Chicago, IL, USA; 10 Cleveland Clinic, Cleveland, OH, USA; 11 Institut Paoli Calmettes, Marseille, France; 12 Padua University School of Medicine, Padua, Italy; 13 Barts Cancer Institute, Queen Mary, University of London, London, UK; 14 Mayo Clinic, Jacksonville, FL, USA; 15 Celgene Corporation, Summit NJ, USA; 16 Celgene Corporation, Boudry, Switzerland; 17 Cologne Cluster of Excellence in Cellular Stress Responses in Aging-associated Diseases (CECAD), Cologne, Germany

## Abstract

Efficacy of lenalidomide was investigated in 103 patients with relapsed/refractory chronic lymphocytic leukemia (CLL) treated on the prospective, multicenter randomized phase-II CLL-009 trial. Interphase cytogenetic and mutational analyses identified *TP53* mutations, unmutated *IGHV*, or del(17p) in 36/96 (37.5%), 68/88 (77.3%) or 22/92 (23.9%) patients. The overall response rate (ORR) was 40.4% (42/104). ORRs were similar irrespective of *TP53* mutation (36.1% (13/36) vs 43.3% (26/60) for patients with vs without mutation) or *IGHV* mutation status (45.0% (9/20) vs 39.1% (27/68)); however, patients with del(17p) had lower ORRs than those without del(17p) (21.7% (5/22) vs 47.1% (33/70); *P*=0.049). No significant differences in progression-free survival and overall survival (OS) were observed when comparing subgroups defined by the presence or absence of high-risk genetic characteristics. In multivariate analyses, only multiple prior therapies (⩾3 lines) significantly impacted outcomes (median OS: 21.2 months vs not reached; *P*=0.019). This analysis indicates that lenalidomide is active in patients with relapsed/refractory CLL with unfavorable genetic profiles, including *TP53* inactivation or unmutated *IGHV*. (ClinicalTrials.gov identifier: NCT00963105).

## key-points

- Overall response rate and survival outcomes are similar in relapsed/refractory chronic lymphocytic leukemia patients treated with lenalidomide irrespective of *TP53* or *IGHV* mutations.
- This suggests that lenalidomide activity may not be affected by loss of functional *TP53* or *IGHV* status.

## Introduction

Single-agent lenalidomide has clinical activity in chronic lymphocytic leukemia (CLL), both in treatment-naive patients,^1, 2^ and in those with relapsed and refractory disease^3, 4, 5, 6^ or unfavorable characteristics.^2, 4, 5^

Recent clinical trials in CLL patients demonstrated that unmutated *IGHV* is associated with unfavorable outcomes with conventional chemotherapy or chemoimmunotherapy regimens.^7, 8, 9^ Multivariate analysis established del(17p), *TP53* mutation or unmutated *IGHV* were each important independent prognostic factors for survival.^7, 9^ *TP53* mutation without del(17p) is also of prognostic importance, with both markers demonstrating independent prognostic significance in multivariate analyses.^10^

Patients with CLL having del(17p) had reduced overall response rate (ORR) and progression-free survival (PFS) in a study involving unselected CLL patients treated in routine clinical practice.^11^ Furthermore, the presence of del(17p) has been associated with significantly inferior outcome in the context of novel, non-cytotoxic treatments, such as ibrutinib.^12^

We investigated the efficacy of lenalidomide in subgroups of relapsed and refractory CLL patients with high-risk genetics and clinical characteristics at baseline.

## Materials and methods

The study design and patient population are described elsewhere.^13^ In brief, patients were randomized 1:1:1 to receive a double-blinded starting dose of oral lenalidomide (5, 10 or 15 mg per day) on days 1–28 of each 28-day treatment cycle. Subject to tolerability, doses were escalated to a maximum of 25 mg per day, with dose modifications applied as required. All patients received appropriate prophylaxis for tumor lysis syndrome and thrombosis. Treatment was continued until disease progression or unacceptable toxicity. Institutional Investigational Review Board of each participating site approved this study, which was conducted according to good clinical practice and the ethical principles outlined in the Declaration of Helsinki. All patients provided written informed consent.

Several exploratory analyses were conducted as part of the trial. Clinical and demographic characteristics of interest were age, disease stage, number of prior treatments, presence of bulky disease or constitutional symptoms and purine analog response status.

Blood samples for *IGHV* and *TP53* mutation analysis, and fluorescence *in situ* hybridization studies for interphase cytogenetic assessment were collected pre-dose on day 1. All genetic analyses were performed centrally (Ulm University, Ulm, Germany, or University of California, San Diego, San Diego, USA), as described.^9, 10, 14^

Descriptive statistics were used to describe continuous demographic and baseline variables for each patient; categorical variables were summarized using frequency tabulations for treatment groups separately and combined. Efficacy analyses were performed on the intention-to-treat population and included all patients with genetic data available. For all efficacy end points, determination of responses (including progression of disease) was based on the investigator's assessment of CLL response data using International Workshop on CLL guidelines for diagnosis and treatment of CLL.^15^ Responses by presence or absence of pretreatment characteristics were compared using logistic regression stepwise selection. Differences were considered significant at the *P*<0.05 level. Logistic regression was done to assess the relationship of patient response (responder vs non-responder) using stepwise selection. The following baseline characteristics were included: relapsed vs refractory to last prior therapy; *IGHV* mutation status; bulky disease; del(17p) and del(11q) status; serum β_2_-microglobulin level; disease stage; and number of prior therapies (<3 vs ⩾3).

### Role of the funding source

Celgene Corporation funded the study. All authors had full access to all data in the study and had final responsibility for the decision to submit for publication.

## Results and discussion

Of 104 patients enrolled, 103 received treatment; baseline characteristics are described elsewhere and the primary results demonstrated that lower starting doses of lenalidomide could facilitate dose escalation, with indication of improved efficacy in patients who escalated to higher doses.^13^

Based on the intent-to-treat safety population (n=103), data on *TP53* mutations, *IGHV* mutational status or del(17p) were available for 96, 89 or 93 patients, respectively. *TP53* mutations were identified in 36 (37.5%) patients, unmutated *IGHV* in 68 (77.5%) patients and del(17p) in 23 (24.7) patients.

Most patients with *TP53* mutations also harbored unmutated *IGHV* (27/36; 75.0%), whereas around half had del(17p) (17/36; 47.2%). In the absence of *TP53* mutation, del(17p) was found in 5/60 (8.3%) patients. A majority of patients with del(17p) also had *TP53* mutations (17/22; 77.3%) or unmutated *IGHV* (16/22; 72.7% Supplementary Table 1). Patients with *TP53* mutations, compared with those without, were more likely to be >65 years (55.6% vs 33.3%), have del(17p) (47.2% vs 8.3%), have Rai high-risk/Binet C disease (55.6% vs 39.3%) or to have a reduced (<1 50 000/mm^3^) platelet count (75.0% vs 45.0% Supplementary Table 1). Patients with unmutated *IGHV* were more likely than patients with mutated *IGHV* to have *TP53* mutation (39.7% vs 20.0%) or bulky disease (45.6% vs 25.0%). Patients with del(17p), compared with those without, were more likely to have *TP53* mutation (36.8% vs 15.0%), Rai high-risk/Binet C disease (45.6% vs 35.0%) or a reduced (<1 50 000/mm^3^) platelet count (77.3% vs 50.0% Supplementary Table 1).

Investigator-assessed ORR was 40.4% (42/104) for all patients (Supplementary Table 2). Median time to first response to lenalidomide for all patients was 3.3 months (range: 1.9–34.9). The median response duration was 22.8 months (range: 16.6–29.3). ORRs for patients with and without *TP53* mutation were 36.1% (13/36) and 43.3% (26/60; *P*=0.526); for patients with and without mutated *IGHV*, ORRs were 45.0% (9/20) and 39.7% (27/68; *P*=0.796). ORR for patients with del(17p) was lower than for those without deletions with borderline significance, using Fisher's exact test (21.7% vs 47.1%, *P*=0.049; odds ratio: 0.31; 95% confidence interval: 0.10 and 0.93). No other significant differences were observed for any other characteristic assessed at baseline.

**Figure 1:**
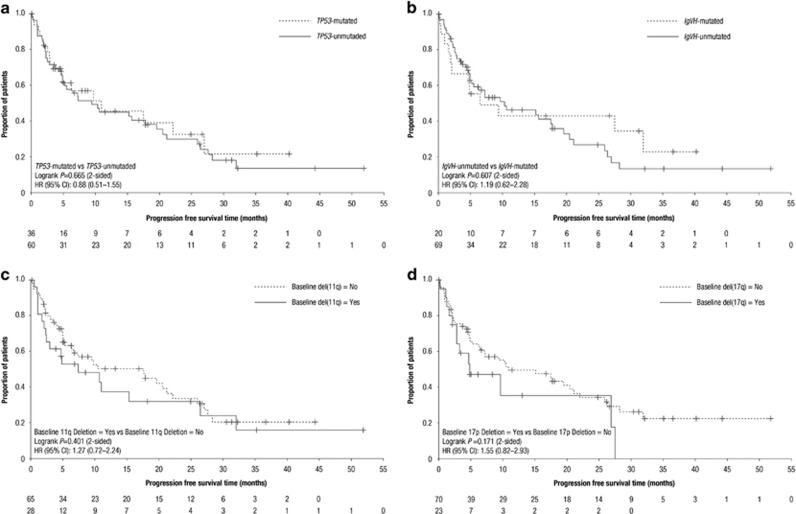
Progression-free survival curves. Patients with and without (**a**) *TP53* mutations; (**b**) *IGHV* mutations; (**c**) del(11q); and (**d**) del(17p).

At a median follow-up time of 24 months, significant survival differences were found between responders and patients with stable disease (median PFS: 26.5 vs 7.2 months, *P*<0.001; median overall survival (OS): not estimable vs 19.8 months; *P*=0.011; Table 1). The median PFS and median OS were 9.7 and 33.0 months, respectively, in the overall population. Median PFS in patients with *TP53* mutations, compared with those without, was short with 11.0 vs 9.5 months (*P*=0.665; Figure 1a); median OS was 19.4 vs 35.4 months (*P*=0.249; Table 1). For patients with mutated vs unmutated *IGHV*, median PFS was 6.5 vs 10.4 months (*P*=0.607; Figure 1b); median OS was 31.9 months vs not estimable (*P*=0.293). In patients with del(11q) vs those without, median PFS was 7.3 vs 17.6 months (*P*=0.401; Figure 1c); median OS was 21.3 vs 35.4 months (*P*=0.435). In patients with del(17p) vs those without, median PFS was 4.9 vs 11.0 months (*P*=0.171; Figure 1d); median OS was 18.9 vs 34.9 months (*P*=0.318; Table 1). Of note, although several of the observed differences between risk groups were sizeable, no significant differences were observed as the study was not powered to detect such differences between risk groups.

Multivariable analyses were performed for PFS and OS including baseline del(11q), del(17p), *TP53* mutation, unmutated *IGHV*, disease stage, relapse/refractory to prior purine analog therapy, baseline β2 microglobulin, bulky disease and number of prior CLL treatments as potential variables. Backward deletion was performed at a significance level of 0.05 and the main effects with *P*-values of ⩽0.05 were retained in the final model and were identified as independent prognostic factors. Regarding PFS, none of the factors were selected into the final model. Regarding OS, only extensive pretreatment (⩾3 lines) significantly impacted outcomes (median OS: 21.2 months vs not reached; hazard ratio: 0.51; 95% confidence interval: 0.28–0.90; *P*=0.019).

Our data reveal that ORR and survival outcomes are similar and relatively poor in relapsed and refractory patients with CLL following lenalidomide treatment irrespective of the presence of *TP53* or *IGHV* mutations, suggesting that lenalidomide activity may not be affected by loss of functional *TP53* or unmutated *IGHV*. Purine analog refractory status and disease stage, both the clinical features associated with high-risk disease, did not appear to impact ORR or survival outcomes following lenalidomide treatment (Supplementary Table 2; Table 1).

In conclusion, our data indicate that a relatively modest lenalidomide activity is seen in relapsed and refractory CLL patients with unfavorable cytogenetic profiles, with ORRs of 36.1% and 39.1% observed in patients with *TP53* mutations and unmutated *IGHV*, respectively. In patients with del(17p), ORR was lower (21.7%) yet still apparent. However, in some patients, these responses were durable as highlighted in the PFS and OS curves (Figure 1). PFS and OS outcomes were similar irrespective of high-risk genetic characteristics. The trial was not powered to detect subtle differences between small subgroups, for example, with del(17p) vs *TP53* mutation. The pleiotropic effects of lenalidomide observed on the tumor microenvironment^16^ or leukemia cell proliferation^17^ and new insights into the various mechanisms of action of lenalidomide are of increasing interest. These insights may provide a rationale for specific combination regimens, including lenalidomide plus ibrutinib, or other agents with distinct mechanisms of action.

## Figures and Tables

**Table 1:** PFS and OS according to pretreatment characteristics^a^

*Characteristic*	N	*Median PFS (months)*	P*-value*^b^	*Median OS (months)*	P*-value*^b^
*All patients*	104	9.7	NE	33.0	NA
Responders	42	26.5	<0.001	NE	0.011
Patients with SD	31	7.2		19.8	

*Binet stage*					NA
Binet stage A	10	3.7		35.4	
Binet stage B	28	15.3		37.7	
Binet stage C	26	27.6		19.7	

*RAI staging system score*					NA
Low-risk disease	5	4.9		20.8	
Intermediate-risk disease	14	19.6		NE	
High-risk disease	21	8.0		28.5	
					
TP53 *mutation*					
Yes	36	11.0	0.665	19.4	0.249
No	60	9.5		35.4	
					
*del(17p)*					
Yes	23	4.9	0.171	18.9	0.318
No	70	11.0		34.9	
					
*del(11q)*					
Yes	28	7.3	0.401	21.3	0.435
No	65	17.6		35.4	
					
IGHV *mutation status*					
Mutated	20	6.5	0.607	31.9	0.293
Unmutated	69	10.4		NE	
					
*Number of prior treatments*					
<3	44	17.6	0.150	NE	0.019
⩾3	60	5.5		21.2	
					
*Bulky disease*					
Yes	45	10.6	0.339	33.0	0.689
No	58	9.7		34.9	
					
*Refractory to purine analogs*					
Yes	44	5.5	0.283	21.3	0.268
No	60	10.4		35.4	

Abbreviations: NA, not applicable; NE, not estimable; OS, overall survival; PFS, progression-free survival; SD, stable disease.

a Based on intent-to-treat population.

b Based on unstratified log-rank test.
