# Supplementary material for: Lenalidomide treatment and prognostic markers in relapsed or refractory chronic lymphocytic leukemia: data from the prospective, multicenter phase-II CLL-009 trial
Source: Blood Cancer J. 2016 Mar 11;6(3):e404–. doi: 10.1038/bcj.2016.9 (PMC4817104; doi:10.1038/bcj.2016.9)
Supplement: Supplementary Table 1 [file bcj20169x1.doc]

# Supplemental Table 1. Baseline characteristics of patients with *TP53* mutation, del(17p), del(11q), and unmutated *IGHV*

| Characteristic | *TP53* mutation | | del(17p) | | del(11q) | | Unmutated *IGHV* | |
| --- | --- | --- | --- | --- | --- | --- | --- | --- |
|  | Yes  (*n* = 36) | No  (*n* = 60) | Yes  (*n* = 22) | No  (*n* = 70) | Yes  (*n* = 28) | No  (*n* = 64) | Yes  (*n* = 68) | No  (*n* = 20) |
| del(17p) | 17 (47.2) | 5 (8.3) | – | – | 3 (10.7) | 19 (29.7) | 16 (23.5) | 3 (15.0) |
| *TP53* mutation* | – | – | 17 (77.3) | 18 (25.7) | 9 (32.1) | 26 (40.6) | 27 (39.7) | 4 (20.0) |
| Unmutated *IGHV* | 27 (75.0) | 40 (66.7) | 16 (72.7) | 46 (65.7) | 20 (71.4) | 42 (65.6) | – | – |
| del(11q) | 9 (25.0) | 18 (30.0) | 3 (13.6) | 25 (35.7) | – | – | 20 (29.4) | 5 (25.0) |
| Age > 65 years | 20 (55.6) | 20 (33.3) | 11 (50.0) | 26 (37.1) | 16 (57.1) | 31 (48.4) | 25 (36.8) | 10 (50.0) |
| Rai III-IV/Binet C disease | 20 (55.6) | 23 (38.3) | 14 (63.6) | 27 (38.6) | 10 (35.7) | 31 (48.4) | 34 (50.0) | 8 (40.0) |
| Bulky^†^ disease | 14 (38.9) | 27 (45.0) | 7 (31.8) | 31 (44.3) | 14 (50.0) | 24 (37.5) | 31 (45.6) | 5 (25.0) |
| ≥3 prior treatments | 18 (50.0) | 38 (63.3) | 11 (50.0) | 41 (58.6) | 20 (71.4) | 32 (50.0) | 35 (51.5) | 16 (80.0) |
| Purine analogue refractory^‡^ status | 13 (36.1) | 24 (40.0) | 11 (50.0) | 28 (40.0) | 12 (42.9) | 27 (42.2) | 27 (39.7) | 9 (45.0) |
| Platelet count < 150,000/mm^3^ | 27 (75.0) | 27 (45.0) | 17 (77.3) | 35 (50.0) | 15 (53.6) | 37 (57.8) | 35 (51.5) | 16 (80.0) |
| Serum β_2_-microglobulin level > 4.0 mg/dL | 22 (61.1) | 38 (63.3) | 14 (63.6) | 43 (61.4) | 16 (57.1) | 41 (64.1) | 40 (58.8) | 13 (65.0) |

All values *n* (%).

*Results for del(17p) were not available for 1 patient with *TP53* mutation.

^†^Bulky disease defined as at least one lymph node > 5 cm.

^‡^Drug refractoriness was defined as failure to achieve at least a partial response to, or disease progression within 6 months of the last dose.
