# Supplementary material for: Lenalidomide treatment and prognostic markers in relapsed or refractory chronic lymphocytic leukemia: data from the prospective, multicenter phase-II CLL-009 trial
Source: Blood Cancer J. 2016 Mar 11;6(3):e404–. doi: 10.1038/bcj.2016.9 (PMC4817104; doi:10.1038/bcj.2016.9)
Supplement: Supplementary Table 2 [file bcj20169x2.doc]

**Supplemental Table 2. ORR according to pretreatment characteristics***

| Characteristic | *N* | ORR | CR | PR | SD | PD | *P*^†^ |
| --- | --- | --- | --- | --- | --- | --- | --- |
| All patients | 104 | 42 (40.4) | 8 (7.7) | 34 (32.7) | 31 (29.8) | 18 (17.3) | NA |
| Binet stage | | | | | | | NA |
| Binet stage A | 10 | 5 (50) | 1 (10) | 4 (40) | 3 (30) | 2 (20) |  |
| Binet stage B | 28 | 13 (46.4) | 4 (14.3) | 9 (32.1) | 5 (17.9) | 7 (25) |  |
| Binet stage C | 26 | 9 (34.6) | 1 (3.8) | 8 (30.8) | 9 (34.6) | 5 (19.2) |  |
| RAI staging system score |  |  |  |  |  |  | NA |
| Low-risk disease | 5 | 2 (40.0) | 0 | 2 (40.0) | 1 (20.0) | 1 (20.0) |  |
| Intermediate-risk disease | 14 | 6 (42.9) | 2 (14.3) | 4 (28.6) | 5 (35.7) | 1 (7.1) |  |
| High-risk disease | 21 | 7 (33.3) | 0 | 7 (33.3) | 8 (38.1) | 2 (9.5) |  |
| *TP53* mutation | | | | | | |  |
| Yes | 36 | 13 (36.1) | 1 (2.8) | 12 (33.3) | 13 (36.1) | 5 (13.9) | .526 |
| No | 60 | 26 (43.3) | 6 (10.0) | 20 (33.3) | 15 (25.0) | 13 (21.7) |
| del(17p) | | | | | | |  |
| Yes | 22 | 5 (22.7) | 0 | 5 (22.7) | 9 (40.1) | 6 (27.3) | .049 |
| No | 70 | 33 (47.1) | 7 (10.0) | 26 (37.1) | 18 (25.7) | 10 (14.3) |
| del(11q) | | | | | | |  |
| Yes | 28 | 10 (35.7) | 0 | 10 (35.7) | 9 (32.1) | 4 (14.3) | .646 |
| No | 64 | 28 (43.8) | 7 (10.9) | 21 (32.8) | 18 (28.1) | 12 (18.8) |
| *IGHV* mutation status | | | | | | |  |
| Mutated | 20 | 9 (45.0) | 2 (10.0) | 7 (35.0) | 4 (20.0) | 5 (25.0) | .796 |
| Unmutated | 68 | 27 (39.7) | 3 (4.4) | 24 (35.3) | 22 (32.4) | 12 (17.6) |
| Number of prior treatments | | | | | | |  |
| <3 | 44 | 17 (38.6) | 2 (4.5) | 15 (34.1) | 15 (34.1) | 6 (13.6) | .841 |
| ≥3 | 60 | 25 (41.7) | 6 (10.0) | 19 (31.7) | 16 (26.7) | 12 (20.0) |
| Bulky disease^a^ | | | | | | |  |
| Yes | 45 | 19 (42.2) | 5 (11.1) | 14 (31.1) | 11 (24.4) | 8 (17.8) | .841 |
| No | 57 | 23 (40.4) | 3 (5.3) | 20 (35.1) | 20 (35.1) | 10 (17.5) |
| Refractory to purine-analog treatment | | | | | | |  |
| Yes | 44 | 15 (34.1) | 4 (9.1) | 11 (25.0) | 11 (25.0) | 10 (22.7) | .314 |
| No | 60 | 27 (45.0) | 4 (6.7) | 23 (38.3) | 20 (33.3) | 8 (13.3) |

All values (except *N* and *P*) are *n* (%).

ORR indicates overall response rate; CR, complete response; PR, partial response; SD, stable disease; PD, progressive disease; NA, not applicable.

*Based on intent-to-treat population.

^†^From Fisher’s exact test.

^‡^Rai low-risk, intermediate-risk, and Binet A and B disease.

^§^Rai high-risk, Binet C disease.

^a^Bulky disease defined as at least one lymph node > 5 cm.
